# Comment on Wunsch et al. The Impact of COVID-19 on the Interrelation of Physical Activity, Screen Time and Health-Related Quality of Life in Children and Adolescents in Germany: Results of the Motorik-Modul Study. *Children* 2021, *8*, 98

**DOI:** 10.3390/children8060520

**Published:** 2021-06-18

**Authors:** Mathilde Kersting, Hermann Kalhoff, Thomas Lücke

**Affiliations:** 1 Research Department of Child Nutrition (FKE), Pediatric University Clinic, Ruhr-University, D-44791 Bochum, Germany; Hermann.Kalhoff@klinikumdo.de (H.K.); luecke.thomas@ruhr-uni-bochum.de (T.L.); 2 Pediatric Clinic Dortmund, D-44137 Dortmund, Germany; 3 Pediatric University Clinic, Medical School, Ruhr-University, D-44791 Bochum, Germany

A recent study concerning the “Impact of COVID-19 on the Interrelation of Physical Activity, Screen Time and Health-Related Quality of Life in Children and Adolescents in Germany” was investigated by Wunsch et al. [1]. To assess adolescents’ subjective health and well-being, the KIDSCREEN-10 index [2] was used, a short-form of the KIDSCREEN-27. Intuitively, one would assume that dietary behaviour is as important a predictor of health-related quality of life (HRQoL) as physical activity and screen time.

A total of 10 dimensions of HRQoL are considered in the basic KIDSCREE‘N 52: physical well-being, psychological well-being, moods and emotions, self-perception, autonomy, parent relations and home life, social support and peers, school environment, social acceptance (bullying), and financial resources. KIDSCREEN-27 and KIDSCREEN-10 consist of five dimensions thereof (Available online: https://www.kidscreen.org/english/questionnaires/kidscreen-52-long-version/, accessed on 22 February 2021).

For nutritionists and paediatricians, nutrition is one further dimension with the potential to have a fundamentally positive impact on quality of life, especially for children. In addition to the direct impact of food on physical health and well-being, we include various positive (hedonic) sensations associated with food intake, among them sensory perceptions such as taste and smell, and increasing feelings of satiety. As is well-known, presentation and visual impact also play an important role in the pleasure of eating, especially for children, and not least the social environment of eating with the family or with friends at school.

The burning glass nature of the Coronavirus pandemic may be an opportunity to re-evaluate the contribution of nutrition to the health-related quality of life in children.

## Data Availability

Data sharing not applicable.
